# Innate Immunity and MASLD

**DOI:** 10.3390/biom14040476

**Published:** 2024-04-13

**Authors:** Moritz Meyer, Julian Schwärzler, Almina Jukic, Herbert Tilg

**Affiliations:** Department of Internal Medicine I, Gastroenterology, Hepatology, Endocrinology & Metabolism, Medical University Innsbruck, 6020 Innsbruck, Austria; moritz.meyer@i-med.ac.at (M.M.); almina.jukic@i-med.ac.at (A.J.)

**Keywords:** MASLD, MASH, innate immunity, cytokines, liver inflammation, hepatology, inflammasome, adipokines

## Abstract

Metabolic dysfunction-associated steatotic liver disease (MASLD) has emerged as the most common liver disease worldwide in recent years. MASLD commonly presents as simple hepatic steatosis, but ~25% of patients develop liver inflammation, progressive fibrosis, liver cirrhosis and related hepatocellular carcinoma. Liver inflammation and the degree of fibrosis are key determinants of the prognosis. The pathophysiology of liver inflammation is incompletely understood and involves diverse factors and specifically innate and adaptive immune responses. More specifically, diverse mediators of innate immunity such as proinflammatory cytokines, adipokines, inflammasomes and various cell types like mononuclear cells, macrophages and natural killer cells are involved in directing the inflammatory process in MASLD. The activation of innate immunity is driven by various factors including excess lipids and lipotoxicity, insulin resistance and molecular patterns derived from gut commensals. Targeting pathways of innate immunity might therefore appear as an attractive therapeutic strategy in the future management of MASLD and possibly its complications.

## 1. Introduction

Metabolic dysfunction-associated steatotic liver disease (MASLD) has recently appeared as the most frequent liver disease worldwide, affecting up to a third of the global population. This is mostly because of the rapid increase in obesity and obesity-related disorders such as type 2 diabetes (T2D) in the past 2 decades around the globe. In many cases, MASLD is a rather inert condition that does not lead to relevant health issues; however, in up to 20–25% of affected individuals, liver inflammation appears, i.e., metabolic dysfunction-associated steatohepatitis (MASH), which drives further liver complications such as advanced fibrosis, liver cirrhosis and finally the development of hepatocellular carcinoma (HCC). What has become highly relevant in the past two decades, however, is the fact that MASLD is strongly associated with substantial extrahepatic disorders such as cardiovascular disease (CVD), inflammatory disorders and an increased rate of extrahepatic malignancies [1]. These extrahepatic complications dominate MASLD-associated mortality, as liver disease with specific hepatic complications only ranks third after CVD and malignancy regarding mortality in MASLD populations. For these reasons, the presence of MASLD has appeared as an important risk factor for affected populations, and therefore MASLD reflects a key feature of human health.

As stated, the presence of simple steatosis without accompanying liver inflammation might not cause relevant liver disease; however, as soon as inflammation evolves in an affected liver, this changes substantially. Tissue-specific inflammation is considered as the driving force in the evolution of organ-specific fibrosis, and it is well established that liver fibrosis defines the prognosis of liver disease [2,3]. While inflammation might not be involved in all cases of MASLD-associated liver fibrosis, evidence is compelling that in most affected individuals, this seems to be the case. Various parts of innate immunity such as several cytokines, adipokines or inflammasomes have been demonstrated in recent years to critically affect to development of MASH. Pro-inflammatory cytokines including various interleukins (IL) and tumor necrosis factor (TNF) are considered prototypic mediators behind an inflammatory liver phenotype. Besides pro-inflammatory cytokines, various adipokines, e.g., adiponectin or leptin, which are mainly produced in adipose tissue, are also crucially linked to obesity and its related disorders. The evolution of inflammation involves numerous other participants, such as inflammasomes [4]. Inflammasomes are critically linked to a proinflammatory cytokine milieu, which arises in an inflamed liver and many diverse inflammatory pathways are activated in parallel. Inflammation-triggering factors in this complex metabolic disease are still the subject of intensive research and it is increasingly accepted that various diverse factors such as pathogenic lipids, insulin resistance or a disturbed gut dysbiosis as observed in MASLD might be of disease-driving importance.

A crucial challenge in the clinical assessment of MASLD patients remains the fact that inflammation so far can only be detected reliably via liver histology, which is not feasible in most affected individuals.

In this article, we will focus on the role of innate immunity in MASLD, although it is acknowledged that besides innate immunity, adaptive immunity has recently appeared as equally important in this disorder [5,6]. Understanding the complex interplay between the different parts of innate immunity in liver inflammation will lead to the development of novel therapeutics in targeting this deleterious condition, affecting nearly a third of the world’s population [7].

## 2. Proinflammatory Cytokines and Their Role in MASLD

More than 20 years ago, it became evident that proinflammatory cytokines contribute substantially to the pathogenesis of MASLD [8,9]. One of the very first clinical studies in MASLD observed the increased expression of TNF and its type 1 receptor, the expression of which positively correlated with the degree of liver fibrosis, and suggested that proinflammatory cytokines affect the progression of disease [10]. Several other studies in the subsequent years have shown that crucial proinflammatory cytokines such as IL-1α/β, IL-6 or various chemokines are highly expressed in patients with inflammation and MASLD. Liver IL-6 expression in patients with MASH also correlated with the degree of inflammation and fibrosis [11]. High-sensitivity C-reactive protein, an acute phase protein which is up-regulated by proinflammatory cytokines, is significantly increased in patients with MASH, and the levels were especially pronounced in patients with advanced fibrosis [12]. The circulating levels of hs-CRP in this study were correlated with intrahepatic CRP mRNA concentrations [12]. An important study by Gadd and colleagues investigated the portal inflammatory infiltrate in various stages of MASLD and cytokine expression [13]. Here, the authors convincingly demonstrated that the cellular infiltrate is composed of cells involved in both innate and adaptive immunity and proinflammatory cytokines such as IL-1β and TNF directing them, which paralleled the degree of the inflammatory infiltrate [13]. Interleukin-1 receptor antagonist (IL-1Ra) is a major antagonist of endogenous IL-1 and reflects an important mechanism of the body in limiting chronic inflammation. Serum IL-1Ra levels also reflect the amount of endogenous inflammation, e.g., in the liver in the case of MASLD, and increased serum levels also mirror the degree of liver inflammation [14]. It is now well established that proinflammatory cytokines are highly expressed in NASH/MASH and their expression affects the degree of fibrosis. Importantly inflammation in this disorder is the driving force of the evolution of fibrosis and fibrosis is the major prognostic factor in MASLD regarding the long-term outcomes of this liver disease [15].

Increased expression of proinflammatory cytokines might also have metabolic consequences, and indeed it has been recognized in the past two decades that proinflammatory cytokines reflect key metabolic messengers. The first proposed “metabolic cytokine” was TNF [16]. Obesity has been shown to be associated with increased synthesis of proinflammatory cytokines not only in visceral and subcutaneous adipose tissue but also in other organs such as the liver or muscles. Gokhan Hotamisligil and colleagues made the first observation that the expression of TNF was increased in various models of obesity and diabetes [17]. Furthermore, they showed that this proinflammatory cytokine might impair insulin signaling, proposing the concept that inflammatory signals impact insulin signaling [18]. The expression of TNF increased in obese premenopausal women, correlated with hyperinsulinemia and decreased after weight loss [19]. Further elegant studies revealed that mice lacking TNF function exhibited improved insulin sensitivity and had lower levels of circulating free fatty acids in diet-induced obesity models [20]. Several studies in the following years demonstrated that targeting TNF by specific monoclonal antibodies improved insulin sensitivity and improved hepatic steatosis, which is frequently observed in obesity and obese mice [21,22]. Despite this striking evidence that TNF is a metabolic cytokine and inflammation is considered of importance in the pathophysiology of insulin resistance, clinical studies using TNF neutralizing strategies have so far not convincingly shown that such an approach significantly impacts metabolic functions including insulin resistance and hyperglycemia [23]. Importantly, placebo-controlled trials with TNF-neutralizing monoclonal antibodies are still not available [16].

Interleukin-1 is another potent pro-inflammatory cytokine which also exhibits various metabolic effects [24]. The IL-1 cytokine family (IL-1F) includes pro- and anti-inflammatory members: the pro-inflammatory members including IL-1α, IL-1β, IL-18, IL-33, IL-36 and IL-38, whereas IL-1Ra or IL-37 have anti-inflammatory action [25]. These mediators exert their specific functions via interaction with specific IL-1 receptors (IL-1R) and IL-1Ra specifically binds to IL-1Rs to prevent IL-1 signaling. Whereas IL-1α is active as a precursor molecule, mature IL-1β needs to be processed by caspase-1, a member of NLR family pyrin domain containing protein 3 (NLRP3). Both IL-1α and IL-1β are highly potent pro-inflammatory mediators triggering the release of other pro-inflammatory cytokines and chemokines, thereby contributing to many acute and chronic inflammatory disorders. In particular, IL-1β has been demonstrated to play a crucial role in MASLD as it activates many diverse liver cells, for example hepatocytes and stellate cells, and plays an important role in the key features of MASLD, such as insulin resistance. Indeed, mice deficient in either IL-1α or IL-1β were protected from liver inflammation in a high-fat diet (HFD) model of MASLD [26]. IL-1α^−/−^ mice also exhibited lower glucose and insulin levels when exposed to an HFD, whereas prolonged treatment with IL-1β worsened insulin signaling in adipocytes [27]. IL-1β knockout mice, after being exposed to an HFD, presented with less hepatic steatosis and almost no adipose tissue inflammation [28]. Furthermore, the administration of IL-1Ra to obese mice improved hepatic steatosis [29]. All these preclinical studies clearly indicate a role for IL-1F members in the propagation of hepatic steatosis, adipose tissue inflammation and regulation of metabolic pathways. We have shown that obese MASLD patients with insulin resistance display very high levels of IL-1β in the adipose tissue (both subcutaneous and visceral adipose tissue), with levels massively exceeding their liver expression, and successful weight loss almost eliminated IL-1β expression in the subcutaneous adipose tissue [30]. Importantly, weight loss also increased the levels of anti-inflammatory IL-1F members such as IL-1Ra and IL-37 in this study. IL-1Ra levels were increased in obese patients with insulin resistance and in patients with MASLD, likely reflecting an inefficient effort of the innate immune system to limit inflammation [14,31]. Interleukin-37 exerts anti-inflammatory and protective functions [32] in many disease models, as IL-37 transgenic mice are protected against obesity-induced inflammation and insulin resistance [33]. Importantly, IL-37 not only suppresses liver inflammation but also decreases liver fibrosis in preclinical experimental models [34]. In summary, IL-1F-member cytokines are crucial players in metabolic inflammation, MASLD and related complications, and targeting these mediators could play a role in clinical management of these patients [35].

## 3. Adipokines: Adipose Tissue-Derived Mediators Contributing to MASLD

Adipokines, especially adiponectin, leptin and many others, are released by healthy and disturbed adipose tissue and have appeared as crucial mediators affecting immunometabolism [36,37]. Although initially believed to be a rather inert organ, the understanding of the adipose tissue has evolved in the past 20 years, and it is now viewed as an endocrinologically and immunologically highly active organ, producing many different mediators collectively termed adipokines. “Adipokine” indicates that such mediators are mainly but not exclusively a product of the adipose tissue, and interestingly adipocytes are also able to synthesize and release many classical immune mediators, i.e., cytokines. Adiponectin has evolved as one of the major products of the adipose tissue, and this section will focus on this pleotropic adipokine. The history of adiponectin began almost 30 years ago when the group of Philipp Scherer identified a 30 kDa protein named adipocyte complement-related protein (Arcp30) via a subtractive cloning approach from 3T3-L1 adipocytes [38]. In 1996, other groups identified the same protein being dysregulated in obesity [39,40,41], and Arita and colleagues proposed the name adiponectin [42]. In the meantime, the literature on this major product of adipocytes exploded (January 2024: 25,485 PubMed articles). Early-adiponectin-knockout mouse studies revealed a phenotype of impaired insulin sensitivity after exposure to an HFD [43,44], and importantly, it was found that the injection of adiponectin into mice improved insulin sensitivity and dyslipidemia [45]. Adiponectin acts via two receptors (AdipoR1 and AdipoR2), thereby eliciting AMP kinase signaling [46]. Indeed, targeted disruption of these receptors also caused insulin resistance and glucose intolerance [47], and a specific agonist (AdipoRon) improved metabolic dysfunction [48].

The success of clinical adiponectin research started with the landmark publication by Arita and colleagues, where they first described that healthy volunteers demonstrate much higher serum concentrations compared to obese subjects, establishing the “adiponectin deficiency” in obesity [42]. Numerous studies followed in many clinical entities, and these clearly showed that low levels of adiponectin can be observed in metabolic dysfunction, including type 2 diabetes [49,50], while certain diseases such as liver cirrhosis, irrespective of etiology, exhibit increased serum levels [51]. In the following years, a plethora of studies indicated that adiponectin exerts anti-inflammatory, anti-apoptotic and anti-fibrotic actions and increases insulin sensitivity. The anti-inflammatory capability of adiponectin correlates with its potential to suppress the synthesis of proinflammatory cytokines and to induce anti-inflammatory cytokines such as IL-10, as we and others have demonstrated [52].

Based on the importance of this adipokine in obesity and obesity-related disorders, many investigators have studied the role of adiponectin in MASLD. Importantly, patients with MASLD not only exhibited reduced serum levels of adiponectin, especially in the case of obesity, but also displayed a lower expression of adiponectin in their livers [53]. In this study, adiponectin protein expression was mainly found in the endothelial cells and decreased AdipoR2 expression correlated with the grade of liver fibrosis [53]. Importantly, massive weight loss, as achieved by means of bariatric surgery, resulted in a significant increase in hepatic and adipose tissue adiponectin mRNA and protein expression [54], which was paralleled by a decrease in hepatic leptin and visfatin expression, thereby generating a more anti-inflammatory adipokine milieu in the body. A large meta-analysis including 28 studies covering MASLD patients demonstrated that patients with MASH exhibited the lowest adiponectin serum levels [55]. Interestingly, even lean MASLD patients show decreased adiponectin levels, a finding which is still not understood and probably reflects a complex interplay between the adipose tissue and the liver [56]. Important for the effects of adiponectin on liver function is the fact that adiponectin can direct Kupffer cells and macrophages towards an anti-inflammatory phenotype. This indicates that adiponectin has detrimental effects on sustaining a healthy liver environment [57]. Adiponectin, as with many other adipokines, can nowadays be considered as part of innate immunity, and these mediators link obesity with related disorders and the immune system. Adiponectin, as a prototypic anti-inflammatory adipokine, can therefore be defined as an important player not only in obesity but also in MASLD and related complications. A thorough discussion of the other adipokines involved in MASLD is beyond the scope of this article.

## 4. Inflammasomes: Key Factors in MASLD

Inflammasomes are prototypic participants in innate immunity and reflect cytosolic multiprotein oligomers, playing a key role in the activation of proinflammatory cytokines such as IL-1β or IL-18. The assembly of inflammasomes allows proteolytic cleavage, maturation, and the secretion of these pro-inflammatory cytokines. The NLRP3 inflammasome, the so far most studied inflammasome involved in metabolic disorders, consists of a protein-nucleotide-binding domain and a leucine-rich repeat NLR family pyrin domain containing 3 protein (NLRP3) or cryopyrin, an apoptosis speck-like protein containing CARD, and the pro-caspase protease caspase-1 [58]. The initial step in an inflammatory reaction is the up-regulation of pro-IL-1β messenger RNA/protein expression. Assembly of the inflammasome complex results in the cleavage of pro-caspase-1 into its active form, caspase-1, which cleaves the pro-IL-1β into its mature and secreted form, IL-1β [59,60]. Inflammasome activation and assembly is directed by various cytosolic pattern recognition receptors (PRR) that respond to either microbe-derived pathogen-associated molecular patterns (PAMPs) or damage-associated molecular patterns (DAMPs).

Especially the NLRP3 inflammasome has been well studied in metabolic inflammation and MASLD. Activity of caspase-1 and IL-1β increase in the adipose tissue after exposure to an HFD or in genetically obese mice [61]. In this study, caspase-1-deficient mice exhibited increased insulin sensitivity. As stated, IL-18 also needs to be processed by caspase-1 to generate mature IL-18 from pro-IL-18. This proinflammatory cytokine is also up-regulated in obese mice and in human obesity [62]. The importance of NLRP3 in obesity and metabolic inflammation has been further demonstrated in a preclinical study where the ablation of NLRP3 improved obesity-related inflammation and metabolic functions [63]. This intervention also resulted in a decrease in IL-18 expression. NLRP3 expression is of importance in preclinical models of MASLD, as a loss of function improves liver inflammation and a gain of function worsens liver disease and associated liver fibrosis [64]. NLRP3 inflammasome activation in myeloid cells plays a role in the progression of murine MASLD by driving a fibrotic phenotype induced by a Western-type diet [65]. The NLRP3 inflammasome seems to be of particular relevance in causing liver fibrosis in metabolic liver disease [66]. NLRP3 activation up-regulates fibrotic markers in hepatic stellate cells and Nlrp3 knock-in mice demonstrate increased liver fibrosis and enhanced collagen production, even independent of the degree of inflammation [67]. However, studies demonstrating a key role of NLRP3 in human MASLD are still rare, and there is a clear need for further studies [68]. This is important as NLRP3 can be antagonized by various drugs such as MCC950, which specifically neutralizes NLRP3 and has been shown to improve MASH pathology, including inflammation and liver fibrosis [69]. Other inflammasome members such as NLRP1 or 6 have not been studied in preclinical MASLD models so far. Overall, there is compelling (preclinical) evidence (although some reports failed to show a convincing protective role of NLRP3 against MASH [70]) that inflammasomes are of crucial importance in MASLD and might especially be relevant in the evolution of fibrosis. The importance of NLRP3 is also proven by the fact that a key product of inflammasome activation (i.e., IL-1β) has been proven to be critical factor in the inflammatory phenotype of this disease. Further studies, both preclinically and clinically, are needed to prove that the inhibition of NLRP3 might finally also benefit patients with this common disease.

## 5. Various Cell Types Involved in Innate Immunity Contribute to This Disease

**Figure 1:**
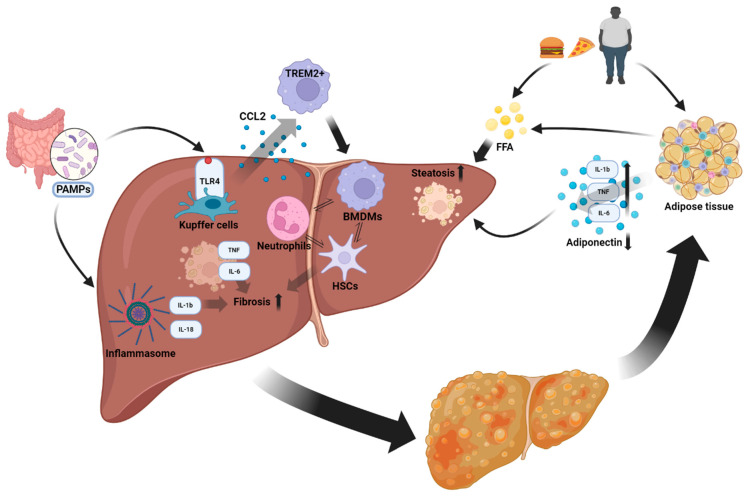
Innate immunity and MASLD: overnutrition and a Western diet fuel obesity, lipotoxicity and adipose tissue inflammation. Cytokines and adipokines derived from the adipose tissue influence hepatic inflammation. Pathogen-associated molecular patterns derived from the gut are sensed by TLR4 and inflammasomes, inducing pro-inflammatory cytokine and chemokine production. BMDMs are recruited to the inflamed liver mainly via chemokines such as CCL2. The crosstalk between innate immune cells, cytokines and various external stimuli induces hepatic inflammation and liver fibrosis. MASH may also promote adipose tissue inflammation, further fueling metabolic diseases.

In addition to numerous paracrine, autocrine, and soluble mediators, inflammation involves a complex and diverse cellular infiltrate (Figure 1). In this article, we will focus on classical cell types directing innate immunity, such as monocytes/macrophages or natural killer (NK) cells, although it is now well known that adaptive immunity (which is not covered in this article) seems to be of equal importance. Of note, the crosstalk between innate and adaptive immunity (as extensively reviewed elsewhere [5]), and also between immune- and non-immune cells such as hepatocytes, promotes liver inflammation in MASLD. For example, lipotoxicity in the hepatocytes induces the release of extracellular vesicles, which promotes immune cell and specifically macrophage infiltration into the liver [71,72,73].

Liver macrophages comprise two different cell types, resident Kupffer cells (KCs) of embryonic origin and monocyte-derived macrophages, which are recruited to the liver mainly via the CCL2/CCR2 axis [74]. Dependent on various stimuli, macrophages can differentiate into a pro- (M1) or an anti-inflammatory (M2) phenotype [74]. During MASLD pathogenesis, M1 polarized macrophages seem to have a disease-driving role, and the activation of M2 KCs induces the apoptosis of M1 polarized KCs, which limits liver disease [75].

KCs sense danger signals including cholesterol crystals and free-fatty acids (FFAs) [76], but also PAMPs originating from a decreased intestinal barrier via, e.g., Toll-like receptor 4, which induces the secretion of pro-inflammatory cytokines and chemokines, promoting a pro-inflammatory hepatic environment [77,78]. KCs can also directly influence MASLD pathogenesis by influencing fatty acid metabolism, and the ablation of a specific KC subtype (CD206^hi^ESAM^+^) reduced hepatic steatosis in HFD-fed mice [79]. Of note, during the progression of MASLD, the liver macrophage composition changes, as resident KCs are replaced by bone marrow-derived macrophages [80,81]. Interestingly, MASH impairs the self-renewal of embryonic KCs, causing their replacement by monocyte-derived KCs which display an increased pro-inflammatory transcriptional profile [82].

In MASLD patients, hepatic crown-like structures, macrophage infiltrates surrounding steatotic hepatocytes, are among the main histopathological findings [83], as similarly observed in the adipose tissue of obese individuals [84]. Recently, single-cell RNA sequencing of human and mouse MASH livers revealed an upregulation of Trem2-expressing macrophages [85], which could serve as a potential treatment target [86], while measuring systemic soluble TREM2 may be a feasible option for non-invasively monitoring MASH severity [87]. Interestingly, MASH has also been shown to promote dysfunction within adipose tissue macrophages, further fueling the vicious cycle between adipose tissue inflammation and MASLD [88].

Hepatic macrophages also induce the recruitment and activation of neutrophils via lipocalin 2 [89]. Neutrophil infiltration is one of the key features of MASLD, and it is suspected to directly promote hepatocyte damage [90], while vice versa, neutrophil infiltration and NETosis are induced by liver injury [91]. The pro-inflammatory effects of neutrophils in MASLD and MASH are mediated via the formation of neutrophil extracellular traps (NETs) [92] and reactive oxygen species [93], and the crosstalk between neutrophils and hepatic stellate cells was shown to amplify hepatic fibrosis in a murine model of MASLD [94].

In patients with MASLD, the grade of steatosis is positively correlated with increased myeloperoxidase expression [95] and an elevated neutrophil to lymphocyte ratio is associated with disease severity in MASLD patients [96]. However, data from rodent models also suggest a role for neutrophils and macrophages in the resolution of hepatic inflammation [97].

Numerous studies have also depicted that NK and NKT cells shape MASLD pathogenesis. An increase in NKT cells, for example, has been found in murine MASH models [98,99], while the absence of NKT cells protected mice from liver fibrosis [99]. Notably, hepatic microbes seem to regulate liver inflammation via NKT cells [100]. Furthermore, an increase in NKT cells was found in the livers of MASLD patients, suggesting a disease-driving role for these innate immune cells [101,102,103]. On the other hand, obesity and MASLD seem to impair NK cell function by inducing cell phenotype changes [104,105].

Notably, ample evidence depicts an anti-fibrotic role for NK cells in non-MASLD/MASH rodent models [106,107,108], while some studies indicate that NK cells may promote MASH [109,110], which might be explained by the different cell phenotypes during health and disease [111].

To summarize, innate immune cells influence MASLD by various means, and newer technologies such as sc-RNA sequencing allow us to gain more and more insights into the immune cell infiltrate of MASLD/MASH. This also allows us to study the different immune cell phenotypes during various stages of this complex and heterogenous disease, potentially revealing promising new therapeutic targets.

## 6. How Does Inflammation Evolve in MASLD?

It remains unclear why almost 75–80% of affected MASLD subjects never develop liver inflammation and associated complications, whereas 20–25% do. In 2010, we proposed a “multiple parallel hits model of MASLD”, suggesting that various parallel hits are needed to initiate and propagate inflammation in MASLD [112]. The above-discussed players in innate immunity have to be activated by various PAMPs and DAMPs to initiate and develop inflammation. Whereas early models of this disease proposed that the presence of hepatic steatosis bacterial components such as endotoxins might be linked with an inflammatory model [113], our model suggested that diverse factors from dietary components to proinflammatory lipids or gut microbial factors might act as PAMPs and DAMPs. Furthermore, inflammation generated in the adipose tissue could further augment liver inflammation. Endoplasmic reticulum (ER) stress reflects another critical pathway involved in MASLD pathogenesis. Therefore, it seems likely that both sterile and non-sterile inflammation contribute to liver inflammation. This is also supported by recent evidence showing that bacterial components, but particularly bacterial DNA (most likely gut-derived), are detectable in the liver of obese subjects, which could also drive this disease [114]. Similar data have also recently been presented in mouse models of obesity and MASLD [100,115]. Dietary proinflammatory components and especially pathogenic lipids may, on the other hand, reflect key driving factors of the sterile component of MASH [116]. What is not discussed but is probably of special importance is the contribution of genetic factors as risk factors for developing MASH and further complications of progressive liver disease.

## 7. Conclusions

Research from the past two decades has convincingly demonstrated that MASLD is a disorder in which innate immunity plays a crucial role. One fascinating aspect is that this disease has appeared as a disorder in which metabolic dysfunction is critically linked to inflammation and immunity, establishing MASLD as a prototypic metabolic-inflammatory disease. Many known features of innate immunity are activated in the livers of MASLD patients and may contribute to the disease phenotype. Importantly, sterile but probably also non-sterile inflammation will contribute to MASH and its complications. It remains a challenge to define which factors besides genetic factors dictate whether a subject develops simple hepatic steatosis or an inflammatory phenotype, i.e., MASH. Some crucial players, such as proinflammatory cytokines, adipokines, inflammasomes and certain cell types involved in innate immunity, have been discussed in this review. However, there remain various other proponents of innate immunity which might be involved in this disease process, such as hepatokines, ER stress or complementary factors and others. Importantly, we have to acknowledge that MASLD is commonly part of metabolic syndrome and therefore part of a systemic disorder, which is reflected by the fact that CVD and malignancies are highly relevant for the final outcome of these patients. Therefore, it seems crucial not only to look at the liver but to consider MASLD as a highly relevant and prevalent systemic disorder.
